# Efficacy and safety of obicetrapib in patients with dyslipidemia: An updated meta-analysis of randomized controlled trials

**DOI:** 10.1016/j.ajpc.2025.101303

**Published:** 2025-09-17

**Authors:** Beatriz Araújo, Giang Son Arrighini, Flávia Queiroga, Ensieh Sadat Mansouri, André Rivera, Wellgner Fernandes Oliveira Amador, Ivo Queiroz, Maria Antônia Costa Cruz Akabane, Leo N Consoli, Milene Vitória Sampaio Sobral, Lucas M. Barbosa, Luciana Gioli-Pereira, Erin D. Michos

**Affiliations:** a Departament of Medicine, Nove de Julho University, São Bernardo do Campo, Brazil; b University of Bologna, Bologna, Italy; c Department of Medicine, Emory University School of Medicine, Atlanta, GA, USA; d Department of Medicine, University of Medical Sciences, Tehran, Iran; e Departament of Medicine, Federal University of Campina Grande, Cajazeiras, Brazil; f University of Wisconsin-Madison, Department of Radiology, WI, United States; g Departament of Medicine, Federal University of Juiz de Fora, Juiz de Fora, Brazil; h Department of Medicine, Federal university of Bahia, Salvador, Brazil; i Departament of Medicine, University of Western, Presidente Prudente, Brazil; j Departament of Medicine, Federal University of Minas Gerais, Belo Horizonte, Brazil; k Hospital Israelita Albert Einstein, São Paulo, São Paulo, Brazil; l Division of Cardiology, Johns Hopkins University School of Medicine, Baltimore, MD, USA

**Keywords:** Obicetrapib, CETP inhibitor, LDL cholesterol, Lipoprotein(a), Dyslipidemia

## Abstract

**Introduction:**

Obicetrapib is a novel cholesteryl ester transfer protein (CETP) inhibitor with promising lipid-lowering effects. While earlier CETP inhibitors have shown inconsistent cardiovascular outcomes and safety concerns, the efficacy and safety of obicetrapib remain under active investigation.

**Methods:**

We systematically searched PubMed, Embase, and Cochrane Central databases for randomized controlled trials (RCTs) comparing obicetrapib versus placebo in adults with dyslipidemia or at high cardiovascular risk. We pooled mean differences (MDs) with 95 % confidence intervals (CI) with a random effects model. We used R software version 4.4.2 for statistical analysis.

**Results:**

We included 7 RCTs comprising 3381 participants, of whom 2151 (63 %) received obicetrapib. The mean age was 64.3 years, and 36 % were women. Compared with placebo, obicetrapib significantly reduced mean LDL-C (MD: -37.21 %; 95 % CI: -41.53 to -32.90; *p* < 0.01; I^2^=64 %), lipoprotein(a) (MD: -37.16 %; 95 % CI: -43.63 to -30.70; *p* < 0.01, I^2^=48 %), apolipoprotein B (MD: -24.65 %; 95 % CI: -28.71 to -20.59; *p* < 0.01; I²=83 %), non-HDL-C (MD: -31.90 %; 95 % CI: -34.81 to -28.99; *p* < 0.01; I^2^=0 %), and triglyceride levels (MD: -7.21 %; 95 % CI: -11.13 to -3.30; *p* < 0.01; I^2^=0 %). Interestingly, obicetrapib also reduced the incidence of new-onset diabetes (RR: 0.88; 95 % CI: 0.80 to 0.97; *p* = 0.01; I²=0 %). In contrast, obicetrapib significantly increased HDL-C (MD: 142.17 %; 95 % CI: 117.56 to 166.78; *p* < 0.01; I^2^=98.3 %), total cholesterol (MD: 11.94 %; 95 % CI: 5.61 to 18.28; *p* = 0.01; I^2^=91 %), and apolipoprotein A1 concentrations (MD: 52.76 %; 95 % CI: 41.87 to 63.66; *p* < 0.01; I²=94 %). There were no significant differences in adverse events.

**Conclusion:**

Among patients with dyslipidemia and/or high cardiovascular risk, obicetrapib significantly reduces LDL-C, lipoprotein(a), apolipoprotein B, and non-HDL-C. No significant differences were observed in adverse events, supporting the favorable safety profile of obicetrapib.

**Figure:**
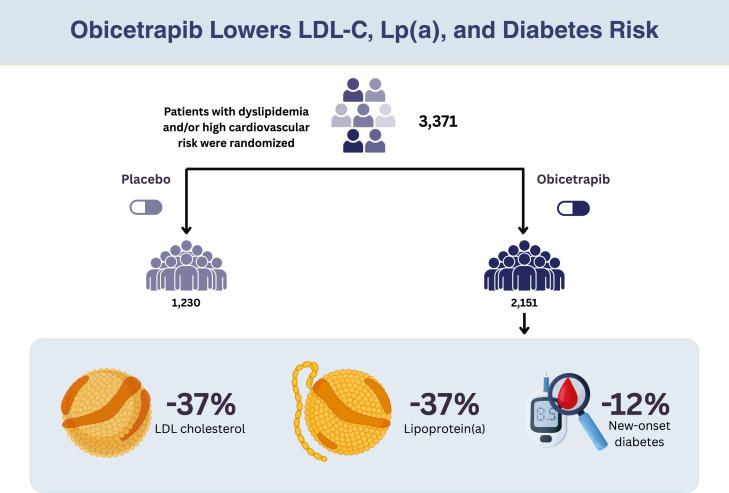
central_illustration.

## 1 Introduction

Lowering low-density lipoprotein cholesterol (LDL-C) is a well-established strategy for reducing the risk of major cardiovascular events [1,2]. Each 40 mg per deciliter reduction in LDL-C is associated with approximately a 22 % decrease in cardiovascular risk [3]. Despite the widespread use of statins and other lipid-lowering therapies, a substantial proportion of individuals remain at elevated risk, often due to persistently high LDL-C levels or other lipid-independent mechanisms of residual cardiovascular risk [[4], [5], [6]].

This clinical gap has driven ongoing interest in the development of novel therapies that can further reduce LDL-C levels when used in combination with statins. Among the most promising strategies are cholesteryl ester transfer protein (CETP) inhibitors, which act by modifying lipid transport and metabolism, thereby reducing atherogenic lipoprotein particles. CETP inhibitors were initially developed on the premise that increasing high density lipoprotein cholesterol (HDL-C) would prevent cardiovascular disease. However, the loss of function mutations in CETP gene do not only lead to increased HDL-C but also lower LDL-C, non-HDL-C, and apolipoprotein B (apoB). Therefore, inhibiting CETP has the potential to lower coronary heart disease risk by lowering concentrations of atherogenic lipoproteins (i.e., LDL-C and apoB), not necessarily by increasing HDL-C.

Unfortunately, early generations of CETP inhibitors were either associated with off-target adverse effects with safety concerns, or had no significant or only modest LDL-C lowering and thus failed to demonstrate cardiovascular benefit, leading to a loss of enthusiasm in this drug class [[7], [8], [9], [10]]. However, obicetrapib, a novel, oral, highly selective CETP inhibitor, has shown encouraging results conferring more potent lowering of atherogenic lipids/lipoproteins without a clear increase in adverse events compared with placebo.

A prior meta-analysis involving only 288 individuals showed a significant reduction in atherogenic lipoproteins with obicetrapib. Since then, three large-scale phase 3 randomized controlled trials (RCTs), the TANDEM, BROADWAY, and BROOKLYN trials, have been published, collectively enrolling over 3000 patients [11]. Therefore, we conducted a systematic review and meta-analysis of RCTs comparing obicetrapib versus placebo in efficacy and safety outcomes.

## 2 Materials & methods

The systematic review and meta-analysis were performed and reported following the Cochrane Collaboration Handbook for Systematic Reviews of Interventions and the Preferred Reporting Items for Systematic Reviews and Meta-Analysis (PRISMA) Statement guidelines [12,13]. The prospective meta-analysis protocol was registered at the International Prospective Register of Systematic Reviews (PROSPERO; CRD420251074053). PRISMA checklists are presented in Supplemental Methods 1 and 2.

### 2.1 Data source and search strategy

We systematically searched PubMed/MEDLINE, Embase, and Cochrane Central Register of Controlled Trials from inception to June 2025. We also used backward snowballing (i.e., review of references and “related articles” sections) to identify relevant texts from articles identified in the original search. Two authors (E.S.M. and G.S.A.) independently screened titles and abstracts and evaluated the articles in full for eligibility based on prespecified criteria, disagreements were resolved in a panel discussion between authors. The search terms included “obicetrapib” AND “TA-8995”.

### 2.2 Study eligibility

There was no restriction concerning the publication date, sample size, follow-up duration, status, or language. Studies were eligible if they (1) were RCTs; (2) enrolled adult patients with dyslipidemia and/or high cardiovascular risk; (3) directly compared obicetrapib with placebo; and (4) reported any prespecified efficacy or safety outcome of interest. For trials that also evaluated a fixed dose combination of obicetrapib with ezetimibe (i.e. ROSE2, TANDEM), only the obicetrapib monotherapy arm was included in the meta-analysis. When multiple timepoints were reported, data from the primary efficacy endpoint were extracted; specifically, day 84 was used for the BROOKLYN and BROADWAY trials.

### 2.3 Outcomes

Our efficacy outcomes were changes from baseline in (1) LDL-C, (2) lipoprotein(a) [Lp(a)], (3) apoB, (4) non-HDL-C, (5) HDL-C, (6) triglycerides, (7) total cholesterol, and (8) apolipoprotein A1 levels. Our safety outcomes included incidence of (1) any adverse event, (2) trial agent adverse event, (3) cardiovascular adverse events, and (4) new onset diabetes.

### 2.4 Statistical analysis

We used the Mantel-Haenszel (MH) random-effects model for all outcomes. We pooled risk ratios (RR) with 95 % confidence intervals (CI) for binary endpoints and weighted mean differences (MD) with 95 % CI for continuous outcomes. All tests were two-tailed, and statistical significance was set at a p-value of <0.05. Means and standard deviations were estimated if necessary. Heterogeneity was assessed using Cochrane’s Q test and Higgins and Thompson’s I^2^ statistics, with *p* ≤ 0.10 indicating statistical significance. We determined the consistency of the studies based on I^2^ values of 0 %, ≤25 %, ≤50 %, and >50 %, indicating no observed low, moderate, and substantial heterogeneity, respectively. We used R software, version 4.5.0, for statistical analysis (R Studio for Statistical Computing, Vienna, Austria).

### 2.5 Subgroup and sensitivity analysis

Prespecified subgroup analyses for change from baseline in LDL-C were performed according to baseline statin status (use versus non-use). Leave-one-out sensitivity analyses were conducted to ensure that the results were not driven by any single study. Additionally, univariate meta-regression analyses were performed to assess whether baseline LDL-C concentrations influenced the magnitude of LDL-C reduction from baseline.

### 2.6 Quality assessment

Quality assessment of RCTs was performed using the Cochrane Collaboration’s tool for assessing risk of bias in randomized trials (RoB-2) by two independent authors (M.A.C.C.A. and M.S.) [14]. Any disagreements were resolved by consensus between authors.

## 3 Results

### 3.1 Study selection and characteristics

**Fig. 1:**
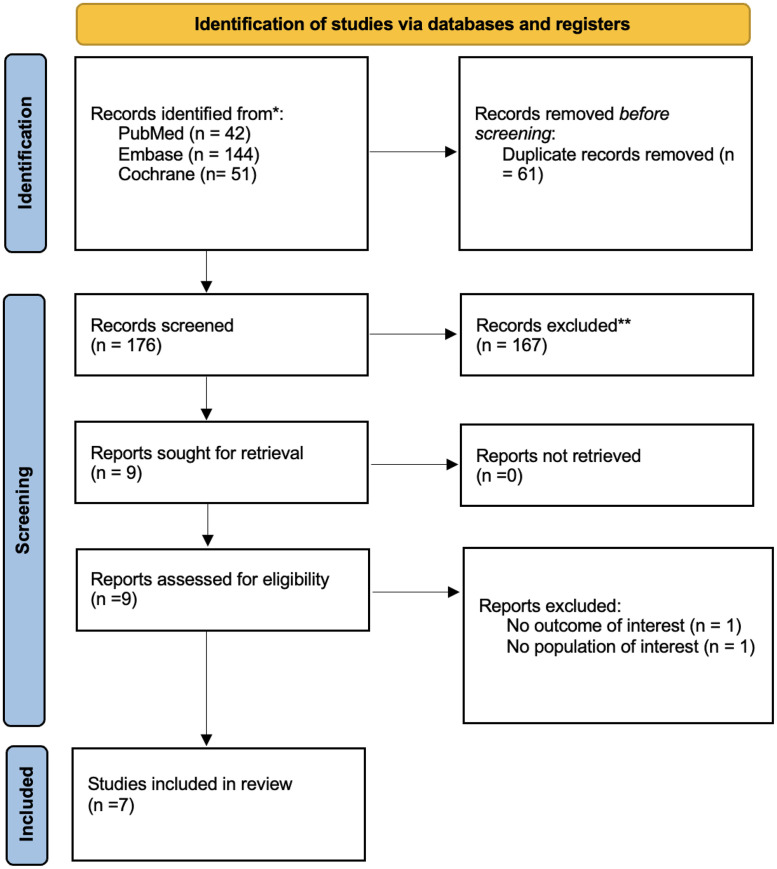
PRISMA flow diagram of study screening and selection.

Our systematic search yielded 237 potential results (Fig. 1). After removing duplicates and ineligible studies, 176 remained for full-text revision. Seven RCTs met the eligibility criteria [[15], [16], [17], [18], [19], [20], [21]]. Overall, 3381 individuals were included, of whom 2151 (63 %) were randomized to receive obicetrapib. Baseline characteristics of the included studies are reported in Table 1.

**Table 1:** Study and baseline patient characteristics.

Study and year	TULIP 2015	ROSE 2022	ROSE2 2023	HARADA-SHIBA 2024	BROADWAY 2025	BROOKLYN 2025	TANDEM 2025
Intervention doses (mg/dl)	1; 2.5; 5; 10	5; 10	10	2.5; 5; 10	10	10	10
Sample Size, (O/P)	35/36	40/40	26/40	26/26	1686/844	236/118	102/102
Follow-up, (months)	3	2	3	2	12	12	3
Age, years (O/P)	66.0/64.4	62.9/61.3	64.8/60.6	62.5/63.3	65.4/65.3	57/56.6	67.0/67.2
Female sex, % (O/P)	22/5.0	37.5/52.5	34.6/35	26.9/23.1	34/33.2	53/55.1	32/50
BMI, kg/m^2^ (O/P)	25.9/26.0	30.8/30.2	29.9/30.8	26.1/25.5	29.4/29.7	NA	NA
Statin use, % (O/P)	Not used	100/100	100/100	100/100	90.9/92.7	NA	84/91
LDL-C, mg/dl (O/P)	135/147	105.5/112	106/115.5	109/110	98.1/98.4	123.4/119.9	100.5/92.8
Lp(a), nmol/L (O/P)	44.4/51.8	29.9/45.3	NA	NA	64/63	NA	64.4/75.2
ApoB, mg/dl (O/P)	100/100	93.5/94	83.25/94	93.5/91.7	91.6/91.9	NA	90.6/85.3

Notes: Data is presented as means. Abbreviations: apolipoprotein B; BMI: body mass index; LDL-C: low density lipoprotein cholesterol; Lp(a): lipoprotein (a); NA: not available; O: obicetrapib; P: placebo.

Among participants with available data, mean age was 64.3 years, and 36 % were women. Baseline LDL-C was 112 mg per deciliter, and baseline Lp(a) was 62 nmol/L. Follow-up durations ranged from 2 to 12 months. With exception of the TULIP trial, which enrolled patients not receiving background statin therapy, all participants were on moderate- or high-intensity statins [20]. All included RCTs were assessed as having low risk of bias (Supplementary Figure 1).

### 3.2 Efficacy outcomes

**Fig. 2:**
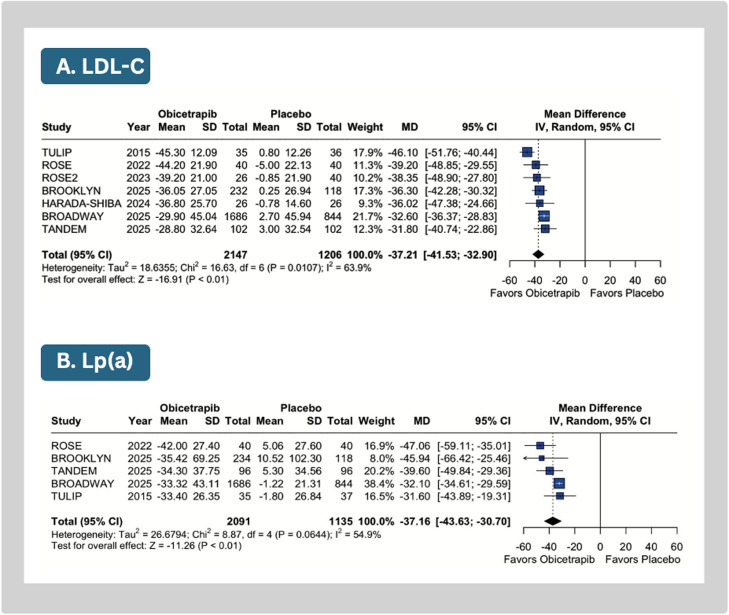
Meta-analysis of the effects of obicetrapib vs placebo on key lipid parameters. Forest plots show percentage reductions in LDL-C and lipoprotein(a). Caption: Forest plots presenting mean differences (MD) with 95 % confidence intervals (CI). Individual study data include means and standard deviations (SD). Abbreviations: CI, confidence interval; SD, standard deviation.

**Fig. 3:**
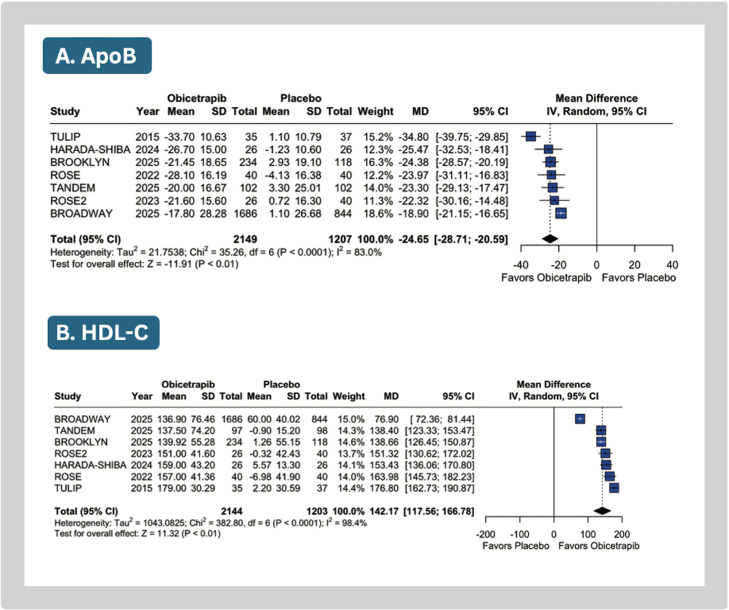
Meta-analysis of the effects of obicetrapib vs placebo on key lipid parameters. Forest plots show reductions in apolipoprotein B levels and an increase in HDL-C. Caption: Forest plots presenting mean differences (MD) with 95 % confidence intervals (CI). Individual study data include means and standard deviations (SD). Abbreviations: CI, confidence interval; SD, standard deviation.

Compared with placebo, obicetrapib significantly reduced mean LDL-C (MD: −37.21 %; 95 % CI: −41.53 to −32.90; *p* < 0.01; I^2^=64 %; Fig. 2A), Lp(a) (MD: −37.16; 95 % CI: −43.63 to −30.70; *p* < 0.01, I^2^=55 %; Fig. 2B), apoB (MD: −24.65 %; 95 % CI: −28.71 to −20.59; *p* < 0.01; I²=83 %; Fig. 3A), non-HDL-C levels (MD: −31.90 %; 95 % CI: −34.81 to −28.99; *p* < 0.01; I^2^=0 %; Supplemental Figure 2A), and triglycerides (MD: −7.21 %; 95 % CI: −11.13 to −3.30; *p* < 0.01; I^2^=0 %; Supplemental Figure 2B).

Obicetrapib significantly increased in HDL-C levels (MD: 142.17 %; 95 % CI: 117.56 to 166.78; *p* < 0.01; I^2^=98.3 %; Fig. 3B), total cholesterol levels (MD: 11.94 %; 95 % CI: 5.61 to 18.28; *p* = 0.01; I^2^=91 %; Supplemental Figure 2C), and apolipoprotein A1 concentrations (MD: 52.76 %; 95 % CI: 41.87 to 63.66; *p* < 0.01; I²=94 %; Supplemental Figure 2D).

### 3.3 Safety outcomes

**Fig. 4:**
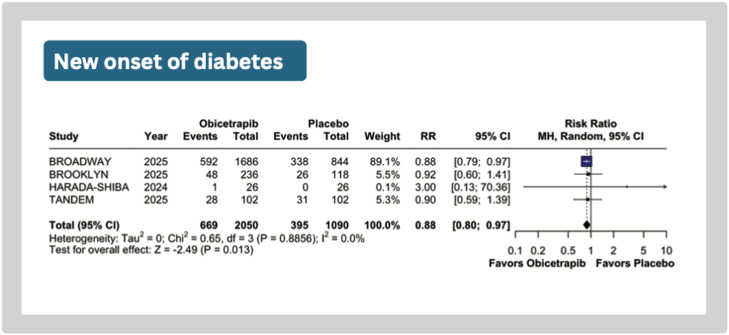
Meta-analysis of the effect of obicetrapib versus placebo on new-onset diabetes. The forest plot shows a reduced risk of new-onset diabetes in the obicetrapib group. Caption: Forest plots presenting risk ratios (RR) with 95 % confidence intervals (CI). Individual study data include events and total number of patients per group. Abbreviations: CI, confidence interval; RR, risk ratio.

Treatment with obicetrapib was associated with a lower risk of new-onset diabetes (RR: 0.88; 95 % CI: 0.80 to 0.97; *p* = 0.01; I^2^=0 %; Fig. 4). There were no significant differences in any adverse events (RR: 0.77; 95 % CI: 0.52 to 1.14; *p* = 0.19; I^2^=92 %), trial agent-related adverse event (RR: 0.93; 95 % CI 0.67 to 1.29; *p* = 0.67; I^2^=10 %), or cardiovascular adverse events (RR: 0.82; 95 % CI: 0.58 to 1.16; *p* = 0.26; I^2^=0 %) (Supplemental Figures 3).

### 3.4 Subgroup and sensitivity analysis

A significant subgroup interaction was observed according to concomitant statin use, with a smaller LDL-C reduction in studies where participants were receiving background statin therapy (MD: −34.35 %) compared to those without statin use (MD: −46.10 %; p_interaction_<0.01; Supplemental Figure 4).

**Fig. 5:**
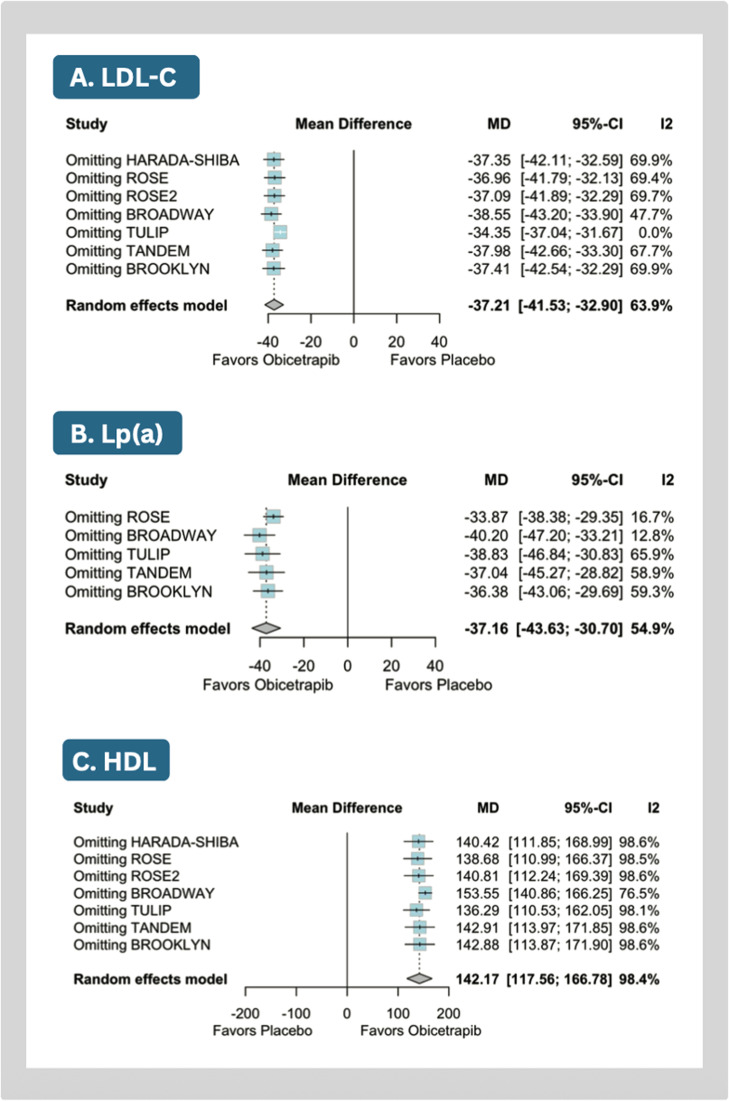
Leave-one-out analysis of the outcomes. Caption: Leave-one-out sensitivity analysis assessing the influence of individual studies on the overall treatment effect. Results indicate the pooled mean difference (MD) with 95 % confidence intervals (CI) after sequential exclusion of each trial. Abbreviations: CI, confidence interval; MD, mean difference.

Omission of the TULIP trial nulled heterogeneity in LDL-C levels but had no impact on Lp(a), and HDL-C levels (Fig. 5). For other endpoints, leave-one-out sensitivity analyses demonstrated consistent results (Supplemental Figures 5). Meta-regression analysis demonstrated a greater LDL-C reduction in studies enrolling individuals with higher baseline LDL-C levels (Supplemental Figure 6).

## 4 Discussion

This systematic review and meta-analysis of 7 RCTs and 3381 patients compared obicetrapib with placebo. We found that obicetrapib significantly reduces key atherogenic lipids, including LDL-C, apoB, non-HDL-C, and Lp(a), with no significant differences in adverse or cardiovascular events compared with placebo. A favorable reduction in new onset diabetes was seen.

The development of CETP inhibitors has historically been hindered by safety concerns and a lack of clear clinical benefits. Torcetrapib, the first CETP inhibitor studied in a large cardiovascular outcome trial, was particularly disappointing: the ILLUMINATE trial was terminated early due to increased all-cause mortality and cardiovascular events despite increasing HDL-C and reducing LDL-C [7]. This was due to an off-target effect of an increase in blood pressure that has not been seen with any of the other CETP inhibitors. Dalcetrapib failed to lower LDL-C and was therefore ineffective in reducing cardiovascular risk [8]. Evacetrapib modestly reduced LDL-C, but its cardiovascular outcome trial was stopped early for futility due to limited statistical power [9]. Anacetrapib, evaluated in the REVEAL trial, showed modest but statistically significant reductions in major cardiovascular events without major safety concerns; nonetheless, it was ultimately not brought to market, reflecting the modest clinical benefit [10].

The magnitude of LDL-C reduction observed with obicetrapib in our meta-analysis is particularly meaningful when compared to established oral lipid-lowering therapies. High-intensity statin regimens typically reduce LDL-C by about 50 %, and moderate-intensity statins by 30–40 % [22]. Ezetimibe, when added to statins, provides an additional 15–25 % LDL-C reduction [23]. Bempedoic acid reduces LDL-C by 24 % when used alone and 18 % when added to a statin [24,25]. Injectable medications, such as the PCSK9 monoclonal antibodies or anti-PCSK9 small interfering RNA therapy, can lower LDL-C by 50–60 %. Despite these options, many high-risk patients fail to achieve guideline-recommended LDL-C targets [4].

Combination therapy is often needed in order to achieve risk-based LDL-C thresholds. Recent phase 2 and phase 3 trials demonstrated that combining obicetrapib with ezetimibe led to substantial additional LDL-C reductions, even in patients already on high-intensity statins, with no major safety signals [18,19]. In the TANDEM trial, the fixed dose combination of obicetrapib and ezetimibe decreased LDL-C by 48.6 % [19].

In our pooled analysis, most participants were receiving moderate- or high-intensity statin therapy. The addition of obicetrapib resulted in an incremental LDL-C reduction of approximately 34 %, exceeding the typical benefit achieved with ezetimibe. Meta-regression analysis suggested a greater relative LDL-C reduction in individuals with higher baseline LDL-C levels, supporting the use of obicetrapib as an early add-on strategy, particularly in patients with marked hypercholesterolemia who are unlikely to reach LDL-C targets with statins alone.

Elevated Lp(a) is now well-recognized as an independent risk factor for atherosclerotic cardiovascular disease [26]. Several novel agents, such as the antisense oligonucleotide pelacarsen and the small interfering RNA therapies of olpasiran, lepodisiran, and zerlasiran, are under investigation and have shown the potential to reduce Lp(a) levels by over 80 % in individuals with markedly elevated Lp(a) concentrations (≥150 nmol/L) [27]. However, these therapies are injectable, likely to be costly, and currently targeted toward a narrow subset of high-risk patients with elevated baseline Lp(a) [28]. In contrast, the obicetrapib trials did not select patients on the basis of elevated Lp(a) level. As such, our analysis included patients with a much lower mean baseline Lp(a) of 62 nmol/L, representing a broader population with modest elevations. Obicetrapib was associated with a 37 % reduction in Lp(a) levels, which is higher than the average reduction of 27 % observed with PCSK9 inhibitors [29]. As an oral agent, obicetrapib may offer a more accessible and cost-effective strategy for addressing residual cardiovascular risk in patients with intermediate Lp(a) levels, for whom no approved therapies currently exist.

Obicetrapib also significantly reduced apoB and non-HDL-C levels, of which both are robust markers of atherogenic particle burden. These findings further support its potential as part of a comprehensive lipid-lowering strategy. Although obicetrapib increased HDL-C levels as anticipated by its mechanism of action, it remains unclear whether this translates into clinical benefit, as prior CETP inhibitor trials have demonstrated that HDL-C elevation alone does not necessarily reduce cardiovascular events. Furthermore, Mendelian (genetic) randomization studies have not found a causal link between HDL-C and cardiovascular risk. Low HDL-C frequently tracks with insulin resistance and elevated triglyceride levels, and thus low HDL-C is a marker of elevated cardiovascular risk in observational studies and used in risk prediction models. However, raising HDL-C with pharmacologic interventions (i.e., niacin and CETP inhibitors) did not translate to cardiovascular benefit and as such HDL-C levels is not a target of preventive strategies. However, it should be noted that HDL-C levels do not adequately reflect HDL particle function such as its role in reverse cholesterol transport and its anti-inflammatory properties [[30], [31], [32]]. Prior therapeutic interventions have targeted HDL-C levels and not metrics of HDL function.

Interestingly, our meta-analysis revealed a 12 % lower risk of new-onset diabetes with obicetrapib compared to placebo. This finding is consistent with previous mechanistic evidence linking CETP inhibition and HDL-C elevation to improved glucose metabolism [33,34]. Both experimental and clinical studies have shown that HDL directly enhances insulin sensitivity and glucose homeostasis, providing a plausible biological mechanism for the reduced incidence of diabetes observed with obicetrapib [34,35]. Importantly, this outcome is a prespecified endpoint in the ongoing PREVAIL cardiovascular outcome trial, which will help confirm whether CETP inhibition can reduce diabetes risk prospectively.

While obicetrapib demonstrated an encouraging safety profile, the relatively short follow-up duration and limited number of events across studies restrict definitive conclusions regarding long-term outcomes. It was reassuring that there were numerically fewer cardiovascular events in the obicetrapib arms of BROOKYLN and BROADWAY than placebo arms, but neither of these one-year trials were powered for cardiovascular events. Although improvements in atherogenic lipids are strongly associated with atherosclerotic cardiovascular disease risk reduction, large-scale cardiovascular outcome trials, such as the ongoing PREVAIL trial (NCT05202509), are necessary to confirm the efficacy of obicetrapib in reducing cardiovascular events.

### 4.1 Limitations

This study has several limitations. First, moderate heterogeneity was observed in our analysis, likely related to differences in baseline lipid levels, background lipid-lowering therapies, and obicetrapib dosing regimens across trials. TULIP trial enrolled participants not receiving statin therapy and as expected, showed a more pronounced LDL-C reduction with obicetrapib. However, in a sensitivity analysis, statistical heterogeneity across trials was eliminated, reinforcing the consistency of our findings. Second, the relatively short follow-up periods across trials limit the ability to assess long-term cardiovascular and safety outcomes. Third, the small number of included studies prevented a formal evaluation of publication bias using Egger’s regression test. Lastly, the absence of patient-level data precluded a more granular assessment of important subgroups and potential effect modifiers, such as baseline Lp(a) and comorbidities, that could influence the efficacy of obicetrapib.

## 5 Conclusion

Among patients with dyslipidemia and/or at high cardiovascular risk, obicetrapib significantly reduced LDL-C, Lp(a), apoB, and non-HDL-C compared with placebo. There were no differences in adverse events between groups.

## Funding

This research did not receive any specific grant from funding agencies in the public, commercial, or not-for-profit sectors.

## Data availability

No original data were generated or analyzed by the authors. All data used in this study are publicly available within the cited publications.

## Previous presentations

None.

## Sources of support

This research did not receive any specific grant from funding agencies in the public, commercial, or not-for-profit sectors.

## Disclaimers

None.

## CRediT authorship contribution statement

**Beatriz Araújo:** Writing – review & editing, Writing – original draft, Validation, Project administration, Methodology, Investigation, Formal analysis, Data curation, Conceptualization. **Giang Son Arrighini:** Project administration, Methodology, Data curation, Conceptualization. **Flávia Queiroga:** Data curation. **Ensieh Sadat Mansouri:** Conceptualization. **André Rivera:** Writing – original draft, Methodology, Data curation. **Wellgner Fernandes Oliveira Amador:** Formal analysis, Data curation. **Ivo Queiroz:** Writing – original draft. **Maria Antônia Costa Cruz Akabane:** Investigation, Data curation. **Leo N Consoli:** Formal analysis. **Milene Vitória Sampaio Sobral:** Investigation. **Lucas M. Barbosa:** Formal analysis. **Luciana Gioli-Pereira:** Supervision, Conceptualization. **Erin D. Michos:** Writing – review & editing, Supervision, Methodology, Conceptualization.

## Declaration of competing interest

The authors declare that they have no known competing financial interests or personal relationships that could have appeared to influence the work reported in this paper.

## Appendix Supplementary materials

Image, application 1
